# An introduction to immunology and immunopathology

**DOI:** 10.1186/1710-1492-7-S1-S1

**Published:** 2011-11-10

**Authors:** Richard Warrington, Wade Watson, Harold L Kim, Francesca Romana Antonetti

**Affiliations:** 1 University of Manitoba, Winnipeg, Manitoba, Canada; 2 Dalhousie University, Division of Allergy, IWK Health Centre, Halifax, Nova Scotia; 3 McMaster University, Hamilton, Ontario, Canada; 4 University of Western Ontario, London, Ontario, Canada; 5 Medical Oncology, Department of Internal Medicine, Tor Vergata Clinical Centre, University of Rome, Rome, Italy

## Abstract

In basic terms, the immune system has two lines of defense: innate immunity and adaptive immunity. Innate immunity is the first immunological, non-specific (antigen-independent) mechanism for fighting against an intruding pathogen. It is a rapid immune response, occurring within minutes or hours after aggression, that has no immunologic memory. Adaptive immunity, on the other hand, is antigen-dependent and antigen-specific; it has the capacity for memory, which enables the host to mount a more rapid and efficient immune response upon subsequent exposure to the antigen. There is a great deal of synergy between the adaptive immune system and its innate counterpart, and defects in either system can provoke illness or disease, such as autoimmune diseases, immunodeficiency disorders and hypersensitivity reactions. This article provides a practical overview of innate and adaptive immunity, and describes how these host defense mechanisms are involved in both health and illness.

## Introduction

Over the past decade, there have been numerous advances in our current understanding of the immune system and how it functions to protect the body from infection. Given the complex nature of this subject, it is beyond the scope of this article to provide an in-depth review of all aspects of immunology. Rather, the purpose of this article is to provide medical students, medical residents, primary-care practitioners and other healthcare professionals with a basic introduction to the main components and function of the immune system and its role in both health and disease. This article will also serve as a backgrounder to the immunopathological disorders discussed in the remainder of this supplement. The topics covered in this introductory article include: innate and acquired immunity, passive and active immunization and immunopathologies, such as hypersensitivity reactions, autoimmunity and immunodeficiency.

## The immune system: innate and adaptive immunity

The immune system refers to a collection of cells and proteins that function to protect the skin, respiratory passages, intestinal tract and other areas from foreign antigens, such as microbes (organisms such as bacteria, fungi, and parasites), viruses, cancer cells, and toxins. The immune system can be simplistically viewed as having two “lines of defense”: innate immunity and adaptive immunity. Innate immunity represents the first line of defense to an intruding pathogen. It is an antigen-independent (non-specific) defense mechanism that is used by the host immediately or within hours of encountering an antigen. The innate immune response has no immunologic memory and, therefore, it is unable to recognize or “memorize” the same pathogen should the body be exposed to it in the future. Adaptive immunity, on the other hand, is antigen-dependent and antigen-specific and, therefore, involves a lag time between exposure to the antigen and maximal response. The hallmark of adaptive immunity is the capacity for memory which enables the host to mount a more rapid and efficient immune response upon subsequent exposure to the antigen. Innate and adaptive immunity are not mutually exclusive mechanisms of host defense, but rather are complementary, with defects in either system resulting in host vulnerability [1-3].

### Innate immunity

The primary function of innate immunity is the recruitment of immune cells to sites of infection and inflammation through the production of cytokines (small proteins involved in cell-cell communication). Cytokine production leads to the release of antibodies and other proteins and glycoproteins which activate the complement system, a biochemical cascade that functions to identify and opsonize (coat) foreign antigens, rendering them susceptible to phagocytosis (process by which cells engulf microbes and remove cell debris). The innate immune response also promotes clearance of dead cells or antibody complexes and removes foreign substances present in organs, tissues, blood and lymph. It can also activate the adaptive immune response through a process known as antigen presentation (discussed later) [1,3].

**Figure 1:**
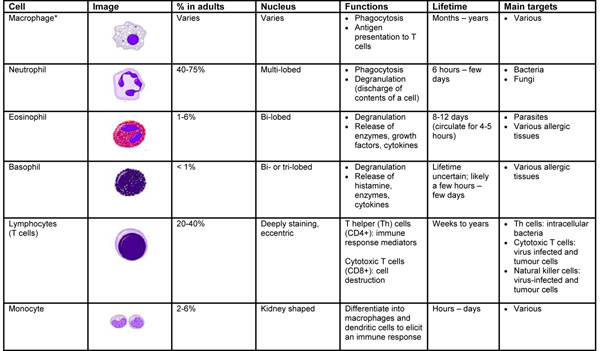
**Characteristics and function of cells involved in innate immunity **[1,3,4]. *Dust cells (within pulmonary alveolus), histiocytes (connective tissue), Kupffer cells (liver), microglial cells (neural tissue), epithelioid cells (granulomas), osteoclasts (bone), mesangial cells (kidney)

Numerous cells are involved in the innate immune response such as phagocytes (macrophages and neutrophils), dendritic cells, mast cells, basophils, eosinophils, natural killer (NK) cells and lymphocytes (T cells). Phagocytes are sub-divided into two main cell types: neutrophils and macrophages. Both of these cells share a similar function: to engulf (phagocytose) microbes. In addition to their phagocytic properties, neutrophils contain granules that, when released, assist in the elimination of pathogenic microbes. Unlike neutrophils (which are short-lived cells), macrophages are long-lived cells that not only play a role in phagocytosis, but are also involved in antigen presentation to T cells. Macrophages are named according to the tissue in which they reside. For example, macrophages present in the liver are called Kupffer cells while those present in the connective tissue are termed histiocytes (see Figure 1) [1].

Dendritic cells also phagocytose and function as antigen-presenting cells (APCs) and act as important messengers between innate and adaptive immunity. Mast cells and basophils share many salient features with each other and both are instrumental in the initiation of acute inflammatory responses, such as those seen in allergy and asthma. Unlike mast cells, which generally reside in the connective tissue surrounding blood vessels, basophils reside in the circulation. Eosinophils are granulocytes that possess phagocytic properties and play an important role in the destruction of parasites that are too large to be phagocytosed. Along with mast cells and basophils, they also control mechanisms associated with allergy and asthma. NK cells (also known as large granular lymphocytes [LGLs]) play a major role in the rejection of tumours and the destruction of cells infected by viruses. Destruction of infected cells is achieved through the release of perforins and granzymes from NK-cell granules which induce apoptosis (programmed cell death) [4]. The main characteristics and functions of the cells involved in the innate immune response are summarized in Figure 1.

Innate immunity can be viewed as comprising four types of defensive barriers: anatomic (skin and mucous membrane), physiologic (temperature, low pH and chemical mediators), endocytic and phagocytic, and inflammatory. Table 1 summarizes the non-specific host-defense mechanisms for each of these barriers.

**Table 1:** Summary of non-specific host-defense mechanisms for barriers of innate immunity [1].

Barrier	Mechanism
**Anatomic**	
Skin	• Mechanical barrier retards entry of microbes • Acidic environment (pH 3-5) retards growth of microbes
Mucous membrane	• Normal flora compete with microbes for attachment sites • Mucous entraps foreign microbes • Cilia propel microbes out of body

**Physiologic**	
Temperature	• Body temperature/fever response inhibits growth of some pathogens
Low pH	• Acidic pH of stomach kills most undigested microbes
Chemical mediators	• Lysozyme cleaves bacterial cell wall • Interferon induces antiviral defenses in uninfected cells • Complement lyses microbes or facilitates phagocytosis

**Phagocytic/endocytic barriers**	
	• Various cells internalize (endocytosis) and break down foreign macromolecules • Specialized cells (blood monocytes, neutrophils, tissue macrophages) internalize (phagocytose), kill and digest whole organisms

**Inflammatory barriers**	
	• Tissue damage and infection induce leakage of vascular fluid containing serum protein with antibacterial activity, leading to influx of phagocytic cells into the affected area

### Adaptive immunity

Adaptive immunity develops when innate immunity is ineffective in eliminating infectious agents and the infection is established. The primary functions of the adaptive immune response are the recognition of specific “non-self” antigens in the presence of “self” antigens; the generation of pathogen-specific immunologic effector pathways that eliminate specific pathogens or pathogen-infected cells; and the development of an immunologic memory that can quickly eliminate a specific pathogen should subsequent infections occur [2]. The cells of the adaptive immune system include: T cells, which are activated through the action of antigen presenting cells (APCs), and B cells.

#### T cells and APCs

T cells derive from hematopoietic stem cells in bone marrow and, following migration, mature in the thymus. These cells express a unique antigen-binding receptor on their membrane, known as the T-cell receptor (TCR), and as previously mentioned, require the action of APCs (usually dendritic cells, but also macrophages, B cells, fibroblasts and epithelial cells) to recognize a specific antigen.

The surfaces of APCs express cell-surface proteins known as the major histocompatibility complex (MHC). MHC are classified as either class I (also termed human leukocyte antigen [HLA] A, B and C) which are found on all nucleated cells, or class II (also termed HLA, DP, DQ and DR) which are found on only certain cells of the immune system, including macrophages, dendritic cells and B cells. Class I MHC molecules present endogenous (intracellular) peptides while class II molecules present exogenous (extracellular) peptides. The MHC protein displays fragments of antigens (peptides) when a cell is infected with a pathogen or has phagocytosed foreign proteins [2,3].

**Figure 2:**
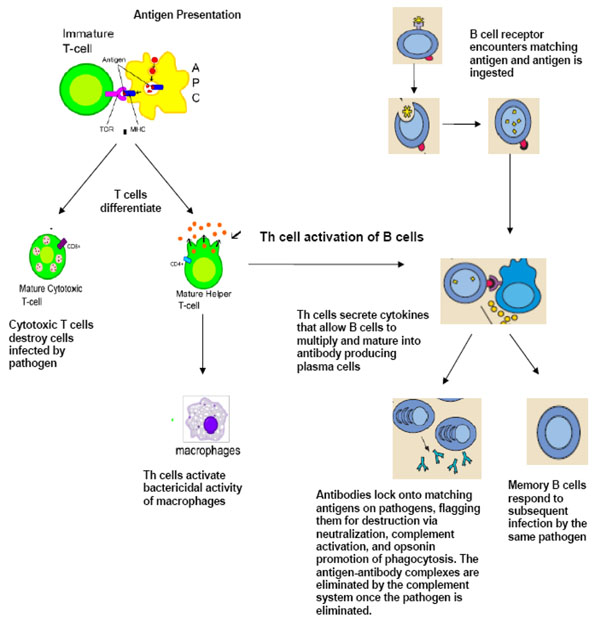
**Adaptive immunity: T-cell and B-cell activation and function.** APC: antigen-presenting cell; TCR: T-cell receptor; MHC: major histocompatibility complex Figure adapted from images available at: http://en.wikipedia.org/wiki/Image:B_cell_activation.png and http://commons.wikimedia.org/wiki/Image:Antigen_presentation.svg

T cells are activated when they encounter an APC that has digested an antigen and is displaying antigen fragments bound to its MHC molecules. The MHC-antigen complex activates the TCR and the T cell secretes cytokines which further control the immune response. This antigen presentation process stimulates T cells to differentiate into either cytotoxic T cells (CD8+ cells) or T-helper (Th) cells (CD4+ cells) (see Figure 2). Cytotoxic T cells are primarily involved in the destruction of cells infected by foreign agents. They are activated by the interaction of their TCR with peptide-bound MHC class I molecules. Clonal expansion of cytotoxic T cells produce effector cells which release perforin and granzyme (proteins that causes lysis of target cells) and granulysin (a substance that induces apoptosis of target cells). Upon resolution of the infection, most effector cells die and are cleared by phagocytes. However, a few of these cells are retained as memory cells that can quickly differentiate into effector cells upon subsequent encounters with the same antigen [2,3].

T helper (Th) cells play an important role in establishing and maximizing the immune response. These cells have no cytotoxic or phagocytic activity, and cannot kill infected cells or clear pathogens. However, they "mediate" the immune response by directing other cells to perform these tasks. Th cells are activated through TCR recognition of antigen bound to class II MHC molecules. Once activated, Th cells release cytokines that influence the activity of many cell types, including the APCs that activate them.

Two types of Th cell responses can be induced by an APC: Th1 or Th2. The Th1 response is characterized by the production of interferon-gamma (IFN-γ) which activates the bactericidal activities of macrophages, and other cytokines that induce B cells to make opsonizing (coating) and neutralizing antibodies. The Th2 response is characterized by the release of cytokines (interleukin-4, 5 and 13) which are involved in the activation and/or recruitment of immunoglobulin E (IgE) antibody-producing B cells, mast cells and eosinophils. As mentioned earlier, mast cells and eosinophils are instrumental in the initiation of acute inflammatory responses, such as those seen in allergy and asthma. IgE antibodies are also associated with allergic reactions (see Table 2). Therefore, an imbalance of Th2 cytokine production is associated with the development of atopic (allergic) conditions. Like cytotoxic T cells, most Th cells will die upon resolution of infection, with a few remaining as Th memory cells [2,3].

A third type of T cell, known as the regulatory T cell (T reg), also plays a role in the immune response. T reg cells limit and suppress the immune system and, thereby, may function to control aberrant immune responses to self-antigens and the development of autoimmune disease.

**Table 2:** Major functions of human Ig antibodies [5].

Ig antibody	Function
IgM	First immunoglobulin (Ig) expressed during B cell development (primary response; early antibody) • Opsonizing (coating) antigen for destruction • Complement fixation

IgG	Main Ig during secondary immune response • Only antibody capable of crossing the placental barrier • Neutralization of toxins and viruses • Opsonizing (coating) antigen for destruction • Complement fixation

IgD	Function unclear; appears to be involved in homeostasis

IgA	Mucosal response; protects mucosal surfaces from toxins, viruses and bacteria through either direct neutralization or prevention of binding to mucosal surface

IgE	Associated with hypersensitivity and allergic reactions • Plays a role in immune response to parasites

#### B cells

B cells arise from hematopoietic stem cells in the bone marrow and, following maturation, leave the marrow expressing a unique antigen-binding receptor on their membrane. Unlike T cells, B cells can recognize free antigen directly, without the need for APCs. The principal function of B cells is the production of antibodies against foreign antigens [2,3].

When activated by foreign antigens, B cells undergo proliferation and differentiate into antibody-secreting plasma cells or memory B cells (see Figure 2). Memory B cells are “long-lived” survivors of past infection and continue to express antigen-binding receptors. These cells can be called upon to respond quickly and eliminate an antigen upon re-exposure. Plasma cells, on the other hand, do not express antigen-binding receptors. These are short-lived cells that undergo apoptosis when the inciting agent that induced the immune response is eliminated.

Given their function in antibody production, B cells play a major role in the humoral or antibody-mediated immune response (as opposed to the cell-mediated immune response, which is governed primarily by T cells) [2,3].

## Antibody-mediated vs. cell-mediated immunity

Antibody-mediated immunity is the branch of the acquired immune system that is mediated by B-cell antibody production. The antibody-production pathway begins when the B cell’s antigen-binding receptor recognizes and binds to antigen in its native form. This, in turn, attracts the assistance of Th cells which secrete cytokines that help the B cell multiply and mature into antibody-secreting plasma cells. The secreted antibodies bind to antigens on the surface of pathogens, flagging them for destruction through pathogen and toxin neutralization, classical complement activation, opsonin promotion of phagocytosis and pathogen elimination. Upon elimination of the pathogen, the antigen-antibody complexes are cleared by the complement cascade (see Figure 2) [2].

Five types of antibodies are produced by B cells: immunoglobulin A (IgA), IgD, IgE, IgG and IgM. Each of these antibodies has differing biological functions and recognize and neutralize specific pathogens. Table 2 summarizes the various functions of the five Ig antibodies [5].

Antibodies play an important role in containing virus proliferation during the acute phase of infection. However, they are not generally capable of eliminating a virus once infection has occurred. Once an infection is established, cell-mediated immune mechanisms are most important in host defense.

Cell-mediated immunity does not involve antibodies, but rather protects an organism through [2]:

• the activation of antigen-specific cytotoxic T cells that induce apoptosis of cells displaying epitopes (localized region on the surface of an antigen that is capable of eliciting an immune response) of foreign antigen on their surface, such as virus-infected cells, cells with intracellular bacteria, and cancer cells displaying tumour antigens;

• the activation of macrophages and NK cells, enabling them to destroy intracellular pathogens; and

• the stimulation of cytokine production that further mediates the immune response.

Cell-mediated immunity is directed primarily at microbes that survive in phagocytes as well as those that infect non-phagocytic cells. This type of immunity is most effective in eliminating virus-infected cells, but can also participate in defending against fungi, protozoa, cancers, and intracellular bacteria. Cell-mediated immunity also plays a major role in transplant rejection.

## Passive vs. active immunization

Acquired immunity is attained through either passive or active immunization. Passive immunization refers to the transfer of *active* humoral immunity, in the form of “ready-made” antibodies, from one individual to another. It can occur naturally by transplacental transfer of maternal antibodies to the developing fetus, or it can be induced artificially by injecting a recipient with exogenous antibodies targeted to a specific pathogen or toxin. The latter is used when there is a high risk of infection and insufficient time for the body to develop its own immune response, or to reduce the symptoms of chronic or immunosuppressive diseases.

Active immunization refers to the production of antibodies against a specific agent *after* exposure to the antigen. It can be acquired through either natural infection with a microbe or through administration of a vaccine that can consist of attenuated (weakened) pathogens or inactivated organisms,

## Immunopathology

As mentioned earlier, defects or malfunctions in either the innate or adaptive immune response can provoke illness or disease. Such disorders are generally caused by an overactive immune response (known as hypersensitivity reactions), an inappropriate reaction to self (known as autoimmunity) or ineffective immune responses (known as immunodeficiency).

### Hypersensitivity reactions

Hypersensitivity reactions refer to undesirable responses produced by the normal immune system. There are four types of hypersensitivity reactions [6,7]:

• Type I: immediate hypersensitivity

• Type II: cytotoxic or antibody-dependent hypersensitivity

• Type III: immune complex disease

• Type IV: delayed-type hypersensitivity

Type I hypersensitivity is the most common type of hypersensitivity reaction. It is an allergic reaction provoked by re-exposure to a specific type of antigen, referred to as an allergen. Unlike the normal immune response, the type I hypersensitivity response is characterized by the secretion of IgE by plasma cells. IgE antibodies bind to receptors on the surface of tissue mast cells and blood basophils, causing them to be “sensitized”. Later exposure to the same allergen, cross-links the bound IgE on sensitized cells resulting in degranulation and the secretion of active mediators such as histamine, leukotriene, and prostaglandin that cause vasodilation and smooth-muscle contraction of the surrounding tissue. Common environmental allergens inducing IgE-mediated allergies include cat-, dog- and horse epithelium, pollen, house dust mites and molds. Food allergens are also a common cause of type I hypersensitivity reactions, however, these types of reactions are more frequently seen in children than adults. Treatment of type I reactions generally involves trigger avoidance, and in the case of inhaled allergens, pharmacological intervention with bronchodilators, antihistamines and anti-inflammatory agents. More severe cases may be treated with immunotherapy.

Type II hypersensitivity reactions are rare and take anywhere from 2 to 24 hours to develop. These types of reactions occur when IgG and IgM antibodies bind to the patient’s own cell-surface molecules, forming complexes that activate the complement system. This, in turn, leads to opsonization, red blood cell agglutination (process of agglutinating or “clumping together”), cell lysis and death. Some examples of type II hypersensitivity reactions include: erythroblastosis fetalis, Goodpasture’s syndrome, and autoimmune anemias.

Type III hypersensitivity reactions occur when IgG and IgM antibodies bind to soluble proteins (rather than cell surface molecules as in type II hypersensitivity reactions) forming immune complexes that can deposit in tissues, leading to complement activation, inflammation, neutrophil influx and mast cell degranulation. This type of reaction can take hours, days, or even weeks to develop and treatment generally involves anti-inflammatory agents and corticosteroids. Examples of type III hypersensitivity reactions include systemic lupus erythematosus (SLE), serum sickness and reactive arthritis.

Unlike the other types of hypersensitivity reactions, type IV reactions are cell-mediated and antibody-independent. They are the second most common type of hypersensitivity reaction and usually take 2 or more days to develop. These types of reactions are caused by the overstimulation of T cells and monocytes/macrophages which leads to the release of cytokines that cause inflammation, cell death and tissue damage. In general, these reactions are easily resolvable through trigger avoidance and the use of topical corticosteroids.

A brief summary of the four types of hypersensitivity reactions is provided in Table 3.

**Table 3:** Types of hypersensitivity reactions [6,7]

Type	Alternate name	Examples	Mediators
I	Allergy (immediate)	Atopy • — Anaphylaxis • — Asthma • — Allergic rhinitis • — Angioedema • — Food allergy	IgE

II	Cytotoxic, antibody-dependent	Erythroblastosis fetalis • Goodpasture’s syndrome • Autoimmune anemias, thrombocytopenias	IgG, IgM

III	Immune complex disease	Systemic lupus erythematosus • Serum sickness • Reactive arthritis • Arthrus reaction	Aggregation of antigens IgG, IgM Complement proteins

IV	Delayed-type hypersensitivity, cell-mediated, antibody-independent	Contact dermatitis • Tuberculosis • Chronic transplant rejection	T cells, monocytes, macrophages

### Autoimmunity

Autoimmunity involves the loss of normal immune homeostasis such that the organism produces an abnormal response to its own tissue. The hallmark of autoimmunity is the presence of self-reactive T cells, auto-antibodies, and inflammation. Prominent examples of autoimmune diseases include: Celiac disease, type 1 diabetes mellitus, Addison’s disease and Graves’ disease [8].

### Immunodeficiency

Immunodeficiency refers to a state in which the immune system's ability to fight infectious disease is compromised or entirely absent. Immunodeficiency disorders may result from a primary congenital defect (primary immunodeficiency) or may be acquired from a secondary cause (secondary immunodeficiency), such as viral or bacterial infections, malnutrition or treatment with drugs that induce immunosuppression. Certain diseases can also directly or indirectly impair the immune system such as leukemia and multiple myeloma. Immunodeficiency is also the hallmark of acquired immunodeficiency syndrome (AIDS), caused by the human immunodeficiency virus (HIV). HIV directly infects Th cells and also impairs other immune system responses indirectly [9,10].

## Conclusion

Innate immunity is the first immunological, non-specific mechanism for fighting against infections. This immune response is rapid, occurring minutes or hours after aggression and is mediated by numerous cells including phagocytes, T cells, mast cells, basophils and eosinophils, as well as the complement system. Adaptive immunity develops in conjunction with innate immunity to eliminate infectious agents; it relies on the tightly regulated interplay between T cells, APCs and B cells. A critical feature of adaptive immunity is the development of immunologic memory or the ability of the system to learn or record its experiences with various pathogens, leading to effective and rapid immune responses upon subsequent exposure to the same or similar pathogens. A brief overview of the defining features of innate and adaptive immunity are presented in Table 4.

**Table 4:** Overview of the defining features of innate and adaptive immunity [1].

	Innate immune system	Adaptive immune system
Cells	Hematopoietic cells: • macrophages • dendritic cells • mast cells • neutrophils • basophils • eosinophils • NK cells • T cells • non-hematopoietic cells • epithelial cells (skin, airways, gastrointestinal tract)	Hematopoietic cells: • T cells • B cells

Molecules	cytokines • complement • proteins and glycoprotein	antibodies (Ig) • cytokines

Response time	immediate	delayed by hours to days

Immunologic memory	none: responses are the same with each exposure	responsiveness enhanced by repeated antigen exposure

There is a great deal of synergy between the adaptive immune system and its innate counterpart, and defects in either system can lead to immunopathological disorders, including autoimmune diseases, immunodeficiencies and hypersensitivity reactions. The remainder of this supplement will focus on the appropriate diagnosis, treatment and management of some of these more prominent disorders, particularly those associated with hypersensitivity reactions.

## Competing interests

Dr Richard Warrington is the past president of the Canadian Society of Allergy & Clinical Immunology and Editor-in-Chief of *Allergy*, *Asthma & Clinical Immunology.* He has received consulting fees and honoraria from Nycomed, CSL Behring and Talecris.

Dr. Wade Watson is a co-chief editor of *Allergy*, *Asthma & Clinical Immunology.* He has received consulting fees and honoraria for continuing education from AstraZeneca, GlaxoSmithKline, King Pharma, Merck Frosst, and Nycomed.

Dr. Harold Kim is the past president of the Canadian Network for Respiratory Care and co-chief editor of *Allergy*, *Asthma & Clinical Immunology*. He has received consulting fees and honoraria for continuing education from AstraZeneca, GlaxoSmithKline, Graceway Pharmaceuticals, King Pharma, Merck Frosst, Novartis, and Nycomed.

Dr. Francesca Antonetti has written articles for Merck Serono.

## References

[B1] Turvey SE Broide DH Innate immunity J Allergy Clin Immunol 2010 125 Suppl 2 S24 32 19932920 10.1016/j.jaci.2009.07.016 PMC2832725

[B2] Bonilla FA Oettgen HC Adaptive immunity J Allergy Clin Immunol 2010 125 Suppl 2 S33 40 20061006 10.1016/j.jaci.2009.09.017

[B3] Murphy KM Travers P Walport M Janeway’s Immunobiology 2007 Seventh New York and London: Garland Science

[B4] Stone KD Prussin C Metcalfe DD IgE, mast cells, basophils, and eosinophils J Allergy Clin Immunol 2010 125 Suppl 2 S73 80 20176269 10.1016/j.jaci.2009.11.017 PMC2847274

[B5] Schroeder HW Cavacini L Structure and function of immunoglobulins J Allergy Clin Immunol 2010 125 Suppl 2 S41 52 20176268 10.1016/j.jaci.2009.09.046 PMC3670108

[B6] Gell PGH Coombs RRA Clinical Aspects of Immunology 1963 First Oxford, England: Blackwell

[B7] Rajan TV The Gell-Coombs classification of hypersensitivity reactions: a re-interpretation Trends Immunol 2003 24 376 379 10.1016/S1471-4906(03)00142-X 12860528

[B8] Castro C Gourley M Diagnostic testing and interpretation of tests for autoimmunity J Allergy Clin Immunol 2010 125 Suppl 2 S238 247 20061009 10.1016/j.jaci.2009.09.041 PMC2832720

[B9] Notarangelo LD Primary immunodeficiencies J Allergy Clin Immunol 2010 125 Suppl 2 S182 194 20042228 10.1016/j.jaci.2009.07.053

[B10] Chinen J Shearer WT Secondary immunodeficiencies, including HIV infection J Allergy Clin Immunol 2010 125 Suppl 2 S195 203 20042227 10.1016/j.jaci.2009.08.040 PMC6151868

